# Around the Hospitals

**Published:** 1897-11-06

**Authors:** 


### AROUND THE HOSPITALS.

[Notice to the Managers of Institutions.—Special space is now reserved for the insertion of notices of hospital meetings and festivals. The Editor requests that all notices may reach The Hospital Office, 28 & 29, Southampton Street, Strand, London, W.C., at least one week before the date of each meeting.]

**City of Dublin Hospital.**—Building and improvements have been the order of the day during the year 1896 at the City of Dublin Hospital. The wards in the Drummond Building were completed; the two observation wards which were animadverted upon by the visiting committee of the Dublin Hospital Fund in 1895 have been improved; a new recreation ground for the patients opened; a new laundry built; the wards, passages, extern departments, corridors, and offices painted in oil instead of distempered; and a new operation theatre has been under consideration. All these matters have resulted in an extraordinary expenditure during the year of about £3,000. In addition the pressure on the resources of the hospital has been increasing, and although the fever wards were closed 1,073 patients were admitted, as against 1,076 in 1895, while 11,963 dispensary and 1,282 accident cases were attended to. In the result the total income of the hospital (including £1,187 from legacies) has been £5,390, while the expenditure has reached £8,434.

**Dundee Royal Infirmary.**—During the year ended May 15th, 1897, there were admitted into the wards of this infirmary 2,687 patients, an increase of 343 on the numbers of the previous year, and a larger number than have been admitted for at any rate six years past. As a consequence of this increase the average number of patients in the hospital daily was 15 more than in 1895-6, or 190 against 175. The largest number of beds occupied on any one day was 235 out of the 286 available, and the lowest number 153. The average stay was less by three days in 1896-7 than in 1895-6, being 24 days against 27 in the latter year. The out-patients numbered 7,905, district patients 6,366, and casualties 1,321. We are glad to note that the revenue showed an increase of £700 over that of the previous year, an increase contributed to by nearly every item of receipts. One exception which we regret to see is a falling off of £78 in the amount of annual subscriptions received. The ordinary expenditure shows an increase of about £680. accounted for by an increase of upwards of £300 on surgery and dispensary expenditure, and about £385 in ordinary repairs. Altogether the accounts, which are kept on the uniform system, show ordinary receipts amounting to £9,001; and legacies and special donations amounting to £15,250, against ordinary expenditure amounting to £10,473, and extraordinary expenditure amounting to £785. To the convalescent branch of the infirmary, the Dundee Convalescent Home, 951 patients were admitted, of whom 533 were patients from the infirmary, and the remainder from the general public.

